# Adult Behavior in Male Mice Exposed to E-Cigarette Nicotine Vapors during Late Prenatal and Early Postnatal Life

**DOI:** 10.1371/journal.pone.0137953

**Published:** 2015-09-15

**Authors:** Dani Smith, Angela Aherrera, Armando Lopez, Enid Neptune, Jonathan P. Winickoff, Jonathan D. Klein, Gang Chen, Philip Lazarus, Joseph M. Collaco, Sharon A. McGrath-Morrow

**Affiliations:** 1 Neurogenetics and Behavior Center, Department of Psychological and Brain Sciences, Johns Hopkins University, Baltimore, Maryland, United States of America; 2 Eudowood Division of Pediatric Respiratory Sciences, Johns Hopkins University School of Medicine, Baltimore, Maryland, United States of America; 3 Division of Pulmonary and Critical Care Medicine, Department of Medicine, Johns Hopkins Medical Institutes, Baltimore, Maryland, United States of America; 4 Julius B. Richmond Center of Excellence, American Academy of Pediatrics, Elk Grove Village, Illinois, United States of America; 5 Division of General Pediatrics, Massachusetts General Hospital and Harvard Medical School, Boston, Massachusetts, United States of America; 6 Department of Pharmacology, Washington State University, Pullman, Washington, United States of America; University of Rennes-1, FRANCE

## Abstract

Nicotine exposure has been associated with an increased likelihood of developing attention deficit hyperactivity disorder (ADHD) in offspring of mothers who smoked during pregnancy. The goal of this study was to determine if exposure to E-cigarette nicotine vapors during late prenatal and early postnatal life altered behavior in adult mice.

**Methods:**

Timed-pregnant C57BL/6J mice were exposed to 2.4% nicotine in propylene glycol (PG) or 0% nicotine /PG once a day from gestational day 15 until delivery. After delivery, offspring and mothers were exposed to E-cigarette vapors for an additional 14 days from postnatal day 2 through 16. Following their last exposure serum cotinine levels were measured in female juvenile mice. Male mice underwent behavioral testing at 14 weeks of age to assess sensorimotor, affective, and cognitive functional domains.

**Results:**

Adult male mice exposed to 2.4% nicotine/PG E-cigarette vapors had significantly more head dips in the zero maze test and higher levels of rearing activity in the open field test compared to 0% nicotine/PG exposed mice and untreated controls. In the water maze test after reversal training, the 2.4% nicotine/PG mice spent more than 25% of time in the new location whereas the other groups did not.

**Conclusion:**

Adult male mice exhibited increased levels of activity in the zero maze and open field tests when exposed to E-cigarette vapor containing nicotine during late prenatal and early postnatal life. These findings indicate that nicotine exposure from E-cigarettes may cause persistent behavioral changes when exposure occurs during a period of rapid brain growth.

## Introduction

Exposure to nicotine during fetal and/or postnatal life in animal and human studies has been associated with changes in adult behavior. [1–3] With the increasing popularity of E-cigarettes among people of child bearing age it is likely that offspring of E-cigarette users will be exposed during pregnancy and childhood to E-cigarette vapors that contain nicotine. Using a murine model we were interested in determining if exposure to E-cigarette vapors that contain nicotine could lead to behavioral changes into adult mice when exposures occurred during a period of rapid brain growth.

Systemic levels of cotinine have been reported in people who use E-cigarettes containing nicotine solutions. [4] In addition detectable cotinine levels have been measured in non-users exposed to E-cigarette vapors that contain nicotine, with cotinine levels similar to that of people exposed to secondhand tobacco smoke. [5] Although studies have reported that exposure to nicotine during early life can lead to changes in adult behavior, little is known whether exposure during early development to E-cigarette vapors that contain nicotine can cause behavioral changes in adults.

In this study mice were exposed to E-cigarette nicotine vapors during late gestation and early postnatal life. The majority of E-cigarette vapor exposure was given during early postnatal life (postnatal day 2–16) to model an infant/child exposure to an E-cigarette user. In the mouse, brain growth during this period of postnatal life correlates with third trimester brain growth in the human. [6] [7] The goal of the study was to determine if exposure to E-cigarette nicotine vapors during a period of rapid brain growth was associated with behavioral changes in adult mice. To this end mice were exposed to E-cigarette vapors containing nicotine from gestational day 15–19 and from postnatal day 2 through 16. Male adult mice then underwent behavioral testing at 14 weeks of age to assess sensorimotor, affective, and cognitive functional domains, with specific procedures carried out in each domain to encompass motor learning and general activity, tests of anxiety-like behaviors, and cognitive flexibility.

## Methods

### Mice

Timed pregnant C57BL/6J mice were obtained from NCI (Bethesda, MD) and all experiments were performed in a C57BL/6J background. The animals were maintained under 12-hour light/dark cycles in a clean environment. Experiments were conducted in accordance with the standards established by the United States Animal Welfare Acts, set forth in NIH guidelines and the Policy and Procedures Manual of the Johns Hopkins University Animal Care and Use Committee. All experiments were approved by the Animal Care and Use Committee at Johns Hopkins University under protocol # MO12M255 approved 7/21/2014. Neonatal mice were euthanized by administration of isofluorane.

### E-cigarette

Joyetech 510-T E-cigarettes were used for all experiments with 510-T tank cartridges, atomizer and battery. The E-cigarette nicotine solutions were obtained from Johnson Creek in 0% and 2.4% nicotine solutions with no flavoring. Refillable cartridges were used.

### E-cigarette chamber and exposure

The E-cigarette (EC) puffs were actuated by a pump (Masterflex 7523–80 L/S Digital Peristaltic Pump Drive) programmed to cycle every 15 seconds. Each actuation was 6 seconds in duration and allowed EC vapors to move through the tubing and fill the chamber. The size of the chamber was 13.5 cm x 9 cm x 8.7cm. Timed pregnant mice from gestational day 15 to 19 were placed in the chamber and exposed to E-cigarette vapors (2.4% nicotine/PG or 0% nicotine/PG, 600 μl) once a day for approximately 20 minutes. Pups were then exposed to E-cigarette vapors (2.4% nicotine/PG or 0% nicotine/PG) once a day from postnatal (PN) day 2 through 16. Postnatal nicotine exposure was approximately 2.1 mg a day. This amount was calculated based on 600 μl of 2.4% nicotine solution being vaped into a chamber which contained a mother and pups. During postnatal exposure the number of pups per litter and the female/male ratio were as follows: The 0% nicotine/PG exposure mice were obtained from 4 litters with 7, 7, 8 and 9 pups respectively in which 42% were males. From these all males (n = 13) were used for behavioral studies. The 2.4% nicotine/PG exposed mice were obtained from 3 litters that contained 6, 7 and 9 pups respectively in which 57% were males. From these all males (n = 8) were used for behavioral studies. Untreated mice were obtained from 2 litters that contained 6 and 5 pups respectively in which 64% were males. From these all males (n = 7) were used for behavioral studies at 14 weeks of age.

### Serum cotinine

Whole blood was obtained from the right ventricle using a 23 gauge needle and spun at 4°C for 10min at 1500 rpm. Serum was isolated and spun again for 5 minutes at 1500 rpm. Serum was then placed in liquid nitrogen and transferred to -80cfvC until analyzed. Blood was drawn from pups starting at 17 hours post exposure to E-cigarette vapors. Cotinine levels in an aliquot of 10 μl sample were first spiked with 5 μl of D3 labeled cotinine standard at 1ppm and then extracted by 30 μl of methanol with 0.1% formic acid by mixing and sitting on ice for 10 minutes. The mixture was then centrifuged at 4°C for 10 minutes, and the supernatant was transferred to a HPLC sample vial and mixed with 155 μl of 5 mM heptafluorobutyric acid (HFBA) for LCMS analysis. The LCMS system used for cotinine quantification consisted of an UPLC system (Waters) and a Xevo G2S QTof (Waters) mass spectrometer. An Aquity UPLC HSS T3 column (2.1x150mm, 1.8 mm) was used for the chromatographic separation. The column oven was set at 30°C. Solvent A was 1 mM HFBA and solvent B was 100% methanol. The LC gradient program was the following: 5% B from 0 to 2 minutes, linear gradient to 20% B from 2 to 3.5 min, 20% B from 3.5 to 6 minutes, linear gradient to 95% B from 6 to 7 min, 95% B from 7 to 9 minutes, and then equilibrating the column with initial conditions for 2 min with a flow rate of 0.4mL/min.

The mass spectrometer was operated in positive MS scan mode, with a source temperature at 120°C, desolvation temperature at 500°C, desolvation nitrogen gas flow rate at 1000L/hour, the cone gas flow rate at 50.0 L/hour, and the sample cone voltage at 30v. TargetLynx (Waters) was used for quantitative analysis. Quantification traces for cotinine and D3-cotinine were m/z 177.1 and 180.12 respectively.

### Behavioral testing

Behavioral testing was done starting at 14 weeks of age. A total of 28 male mice underwent testing (0% nicotine/PG, n = 13, from four litters and 2.4% nicotine/PG, n = 8, from three litters, untreated, n = 7 from two litters). Food and water were available ad libitum. The vivarium was maintained at 25° C and on a 12:12 hour light:dark cycle with lights on at 0700 h and all testing occurred between 0900h–1500h. The following behavioral tests were performed: **Rotarod**: The rotarod (Accuscan, Columbus, OH) consisted of a semi-enclosed chamber containing a beam (Ø = 3 cm, length = 5 cm) suspended above the floor. A mouse was placed on the beam in the orientation opposite to that of its rotation so that forward locomotion is necessary for fall avoidance. Over a 2-minute trial, the rotarod gradually accelerated without jerks from 0–40 rpm. Latencies for the mice to fall from the rod were automatically recorded by a computer. Each mouse was given 4 trials with a 10-minute intertrial interval (ITI) on each of the 3 consecutive days. **Elevated zero maze**: The elevated zero maze (Med Associates, St Albans, VT) consisted of a circular platform, equally divided into 4 quadrants and elevated above the floor: 2 quadrants on opposite sides were enclosed by walls and the other 2 quadrants were open and bordered by a lip. The mouse was placed at one closed arm entrance and allowed to move freely for 5 minutes. Behavior maze was monitored by an overhead mounted camera and tracking system. **The light–dark box test**: The light-dark test was carried out in a chamber (20.3 x 20.3 cm, Accuscan, Columbus, OH) mounted within sound attenuating shells. The chamber was divided into two compartments separated by a partition with a small opening allowing the mouse the pass through. One compartment was lighted with white light while the other was a smaller, opaque, and dark. Behavior was monitored via a grid of invisible infrared light beams mounted on the sides of the walls of the arena. Data was collected and analyzed via VersaMax Analyzer software (Accuscan, Columbus, OH). Each session started by placing the mouse in the dark compartment and allowing it to move freely for 5 minutes. **The open field test**: All procedures were carried out in a square open field chamber (40.6 x 40.6 cm, Accuscan, Columbus, OH) mounted within sound attenuating shells. Behavior was monitored via a grid of invisible infrared light beams mounted on the sides of the walls of the arena. Data was collected and analyzed via VersaMax Analyzer software (Accuscan, Columbus, OH). To examine activity levels and habituation, mice were exposed to the test chambers for 30 minutes on each of two consecutive days. To begin a session, each mouse was placed in the center of the chamber and allowed to move about freely. **The Morris water maze task with reversal learning:** The water maze used for the spatial navigation task consisted of a circular tank (Ø = 1.5m), filled with water between 23–25°C and rendered opaque by the addition of non-toxic white paint. The pool was surrounded by a black curtain containing an arrangement of spatial cues of various shapes and sizes affixed on a white background. Mice were trained to locate an escape platform positioned 1 cm below the surface of the water. This test was carried out in 3 phases: **Phase 1:** Place training to a hidden location, on days 1–4, each mouse had 3 training trials per session, 2 sessions per day separated by 3 hours. In each session, the mouse was placed in one of the 4 start locations, equally spaced around the perimeter of the tank; start locations varied between trials. The mouse was allowed to swim for 60 seconds or until the platform was located. If the platform was not located within 60 seconds, the mouse was placed on the platform by the experimenter for 10 seconds and then placed in a home cage under a heat lamp for a 5 minute ITI. On day 5, a probe session was performed where in a 60 second trial the platform was made unavailable by retracting for the first 30 seconds and then made available for the last 30 seconds by raising it to its original position. The first 30 seconds of the probe trial was used to test the development of spatial bias in searching for the escape platform and the last 30 seconds to allow the mouse to escape. **Phase 2: Reversal Learning:** On days 6 and 7, the platform was relocated to the quadrant opposite that on days 1–4 and mice were trained to the new location. To monitor development of spatial bias for new location, 30-second probe trials were performed at the beginning of day 7 (before daily place training) and day 8. **Phase 3: Cue training:** On day 8, each mouse was given cue-training trials (2 sessions of 2 trials) in which the platform was visible (1 cm above water surface). Each trial followed the same procedure as the Place training phase, except that the location of the escape platform varied between trials and was placed in one of the 2 quadrants not used during Phase 1 and 2.

### Statistics

Statistical analyses were run in SYSTAT 12 (SYSTAT software, Inc., San Jose, CA). The elevated zero maze was analyzed with a One-way ANOVA with Treatment (0% Nicotine, 2.4% Nicotine, Room Air Control) as the factor. Rotarod data, open field, and water maze data was analyzed with a Two-way ANOVA with repeated measures. For the rotarod, open field, and rearing data, Treatment (0% Nicotine, 2.4% Nicotine, Room Air Control) was the between factor and Day was the within factor; for open field; for the water maze training data, Treatment (0% Nicotine, 2.4% Nicotine, Room Air Control) was the between factor and Session was the within factor, and for water maze probe data, Treatment (0% Nicotine, 2.4% Nicotine, Room Air Control) was the between factor and Quadrant was the within factor. An alternate analysis of data was performed using nested mixed models, (S1 Text. Analysis using Nested Mixed Modeling).This method was done to account for litter-based effects and to provide estimates of variance due to litter.[8]

## Results

### Weights and plasma cotinine levels

**Fig 1:**
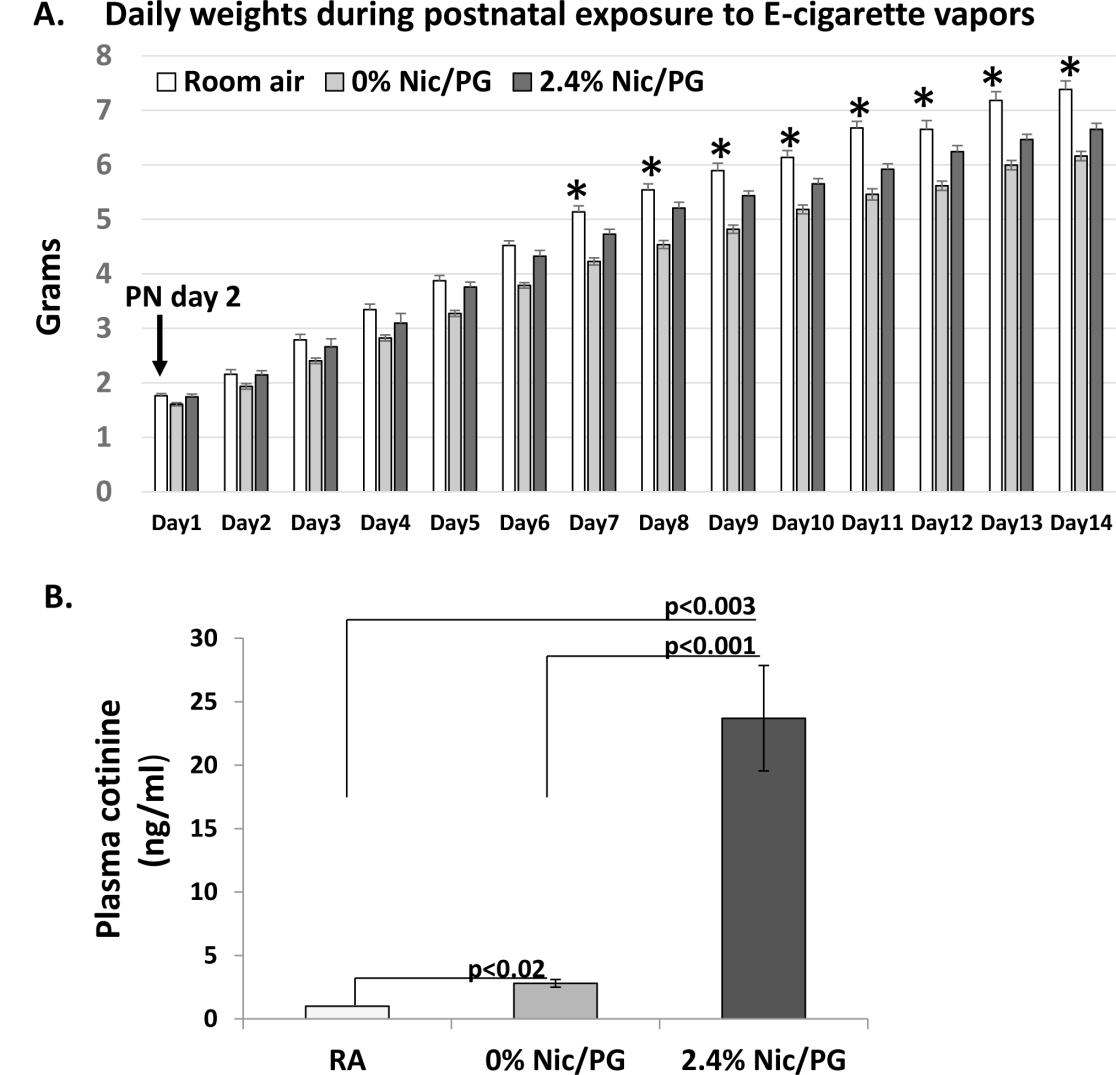
(A) Daily weights during 14 days of postnatal exposure to E-cigarette vapors starting at PN day 2. From 7–14 days of postnatal exposure, pups exposed to 0% nicotine/PG or 2.4% nicotine/PG E-cigarette vapors had significantly lower weights compared to untreated mice (RA) (* p< 0.02). Mice exposed to 0% Nic/PG E-cigarette vapors were significantly smaller than 2.4% Nic/PG and room air mice throughout the exposure (p<0.03, error bars represent standard error of the means) (n = 11–31). (B) Serum cotinine levels from female pups exposed to 14 days of 2.4% Nic/PG or 0% Nic/PG E-cigarette vapors or room air controls (n = 4–7, error bars represent standard deviations).

On postnatal (PN) day 2 following delivery, pups and their mothers were exposed to 14 days of E-cigarette vapors. On the first day of postnatal exposure the mean weight of pups exposed to 0% nicotine/ PG from GD 15–19 was significantly less than 2.4% nicotine/PG or age-matched untreated control pups. At 7 days of postnatal exposure to E-cigarette vapors, the mean weight of the 2.4% nicotine/PG exposed mice was significantly less than age-matched untreated controls and remained so throughout the 14 days of E-cigarette vapor exposure (Fig 1(A)). The mean weight of the 0% nicotine/PG mice also remained significantly less than the untreated or 2.4% nicotine/PG exposed mice throughout the postnatal exposure.

The nicotine exposure was approximated at 2.1 mg/day in pups exposed to 2.4% nicotine vapors. This amount did not include exposure from other sources such as breastmilk or contact with nicotine on fur. As a biomarker for systemic nicotine absorption, serum cotinine levels were measured from female pups at the end of the 14 days of postnatal exposure and within the first half-life of cotinine. The mean serum cotinine levels in the 2.4% nicotine/PG exposed mice was 23.7±4.2 ng/ml. Mean serum cotinine levels in 0% nicotine/PG mice and untreated control mice were 2.8±0.3 ng/ml and 1.0±0.001ng/ml respectively (Fig 1(B)). The male mice in each group were weaned at 3 weeks of age and underwent behavioral testing at 14 weeks of age.

### E-cigarette vapor exposure and behavioral testing in adult male mice

**Fig 2:**
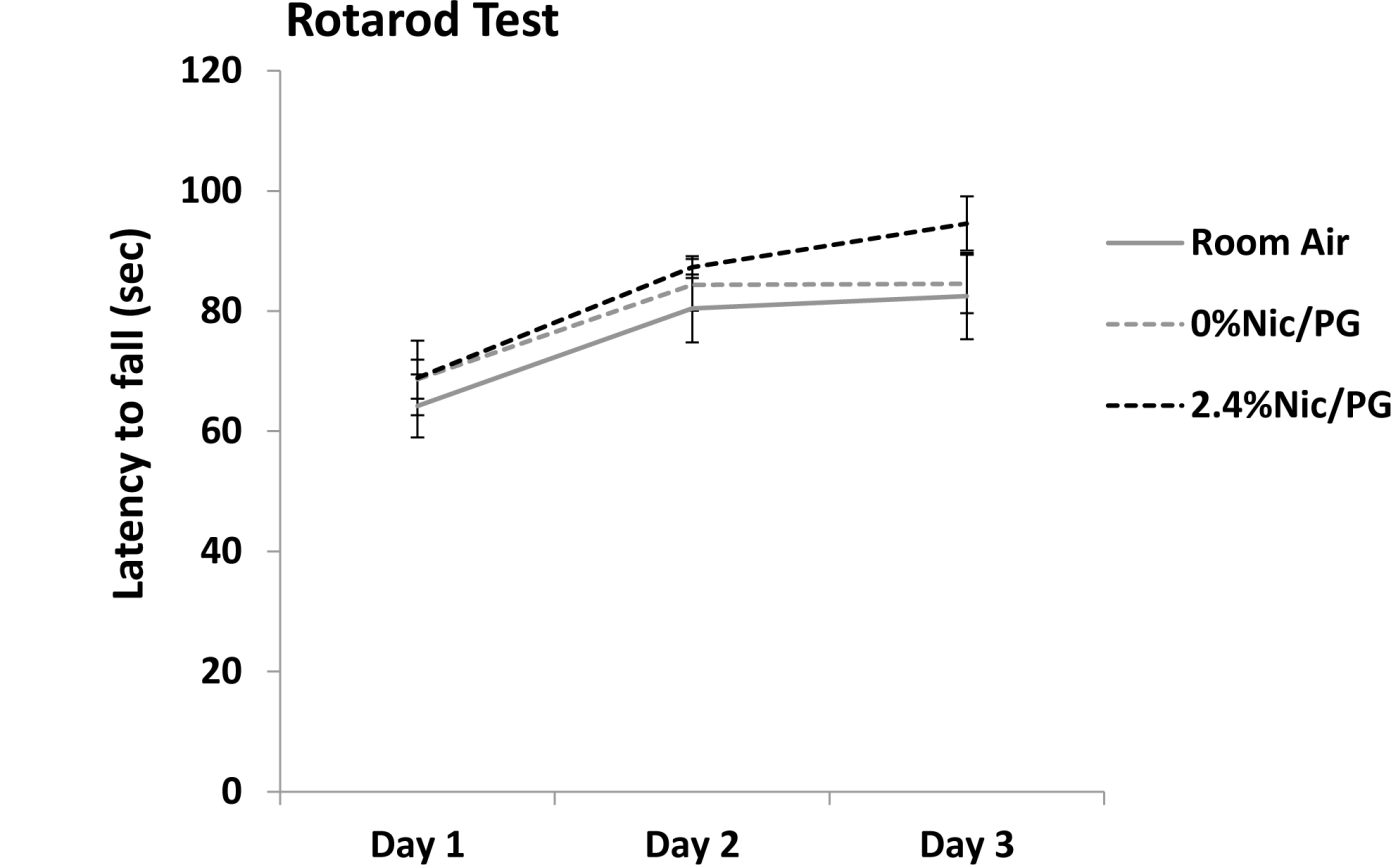
Rotarod Test. Mean latency to fall during the three training sessions. There was no difference between any of the treatment groups from day 1 through day 3. As training progressed all groups of mice demonstrated increased latencies before falling which was confirmed by ANOVA, which yielded a main effect of Day (p < 0.001). (n = 7–13)

Male mice underwent behavioral testing at 14 weeks of age. No difference in mean weights were found between the untreated (26.41± 2.35 grams) and 2.4% nicotine/PG exposed mice (27.95 ± 2.38) or 0% nicotine/PG exposed mice (25.34± 1.85). However there was a modest but significant difference in weights between the 2.4% nicotine/PG and 0% nicotine/PG mice (p<0.01). On the rotarod test no differences were found between the groups of mice tested. All three groups were able to maintain balance on the rod with an increased latency to fall as training progressed (Fig 2). This was confirmed by ANOVA, which yielded a main effect of Day (F_(2,50)_ = 21.524, p < 0.001), but not of Treatment or Day x Treatment interaction.

**Fig 3:**
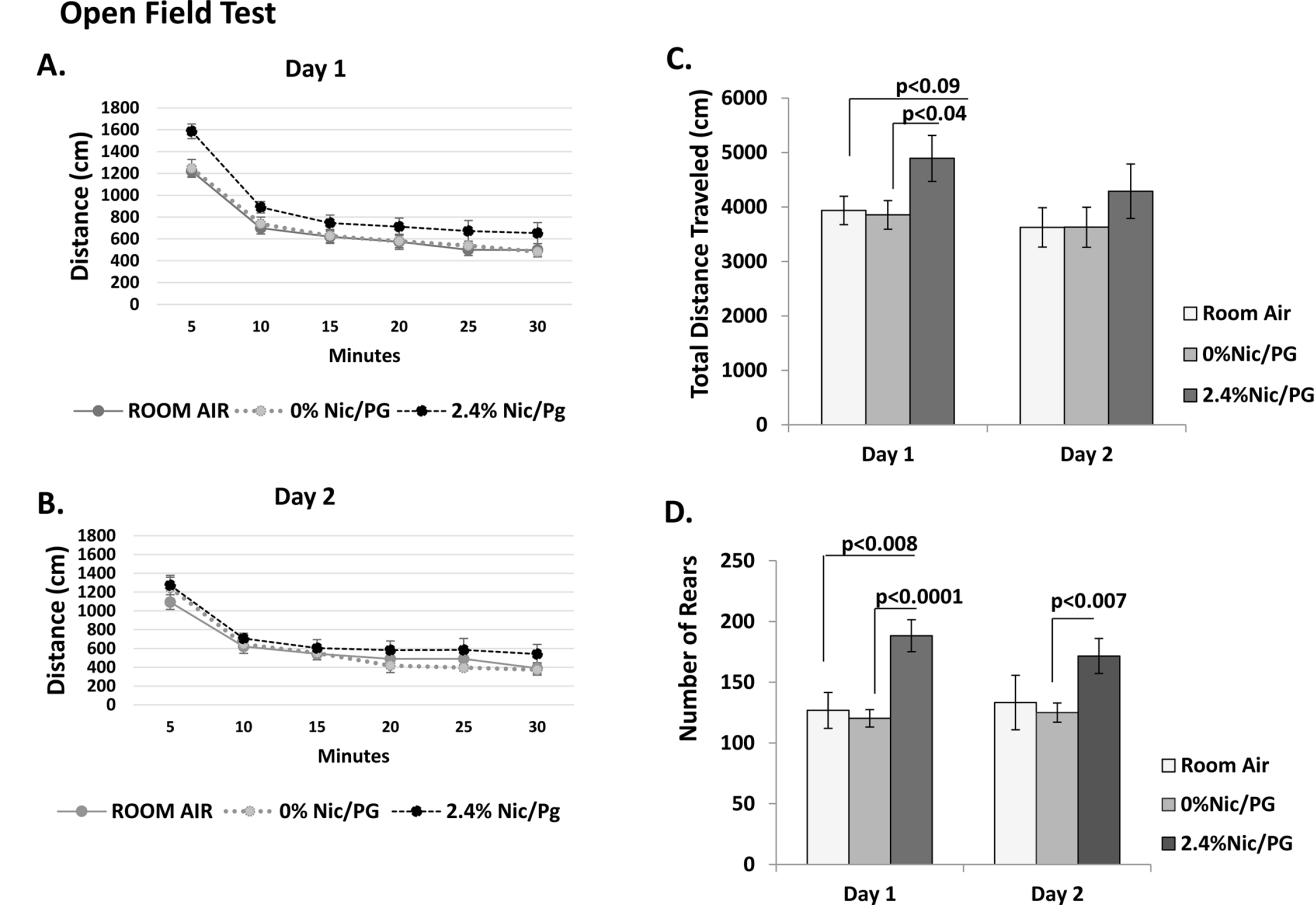
Open Field Test. (A-B) There was a main effect of Day (F_1,25_ = 4.994, p = 0.035). There was no effect of Treatment or Day x Treatment interaction. (C) *t*-tests were used to compare distance travelled on Day 1 between the 2.4% nicotine/PG exposed mice and each of the other two groups. Mice exposed to 2.4% nicotine/PG travelled significantly greater total distances than mice exposed to 0% nicotine/PG (t_19_ = 2.205, p<0.04) and tended to travel a greater distance than mice exposed to room air (t_13_ = 1.857, p<0.09). (D) Rearing behavior was significantly increased in mice exposed to 2.4% nicotine/PG compared to the other groups. This was confirmed in a significant main effect of Treatment (F_1,25_ = 7.438, p = 0.003) in the absence of an effect of Day or Day x Treatment interaction. Analysis of each individual day revealed mice exposed to 2.4% nicotine/PG had a greater number of rears than mice exposed to 0% nicotine/PG on both days (t test p<0.0001, Day1; p<0.007, Day 2), and also a greater number of rears than mice exposed to room air on Day 1 (p<0.008). (n = 7–13)

In the open field test, a test designed to assess general activity levels, gross locomotor activity and exploration habits in mice over a two day testing period, there was a main effect of Day (F_1,25_ = 4.994, p = 0.035) **(** Fig 3A and 3B). There was no effect of Treatment or Day x Treatment interaction. Given the possibility that Day 2 behavior was influenced by Day 1 behavior, *t*-tests were used to compare distance travelled on Day 1 between the 2.4% nicotine/PG exposed mice and each of the other two groups, (Fig 3(C)). Mice exposed to 2.4% nicotine/PG travelled significantly greater total distances than mice exposed to 0% nicotine/PG (t_19_ = 2.205, p<0.04) and tended to travel a greater distance than mice exposed to room air (t_13_ = 1.857, p<0.09).

Rearing behavior was increased significantly in mice exposed to 2.4% nicotine/PG compared to the other groups. There was a significant main effect of Treatment (F_1,25_ = 7.438, p = 0.003) in the absence of an effect of Day or Day x Treatment interaction. Analysis of each individual day revealed mice exposed to 2.4% nicotine/PG had a greater number of rears than mice exposed to 0% nicotine/PG on both days (*t*- test p<0.0001, Day1; p<0.007, Day 2), and also a greater number of rears than mice exposed to room air on Day 1 (p<0.008,Fig 3(D)).

**Fig 4:**
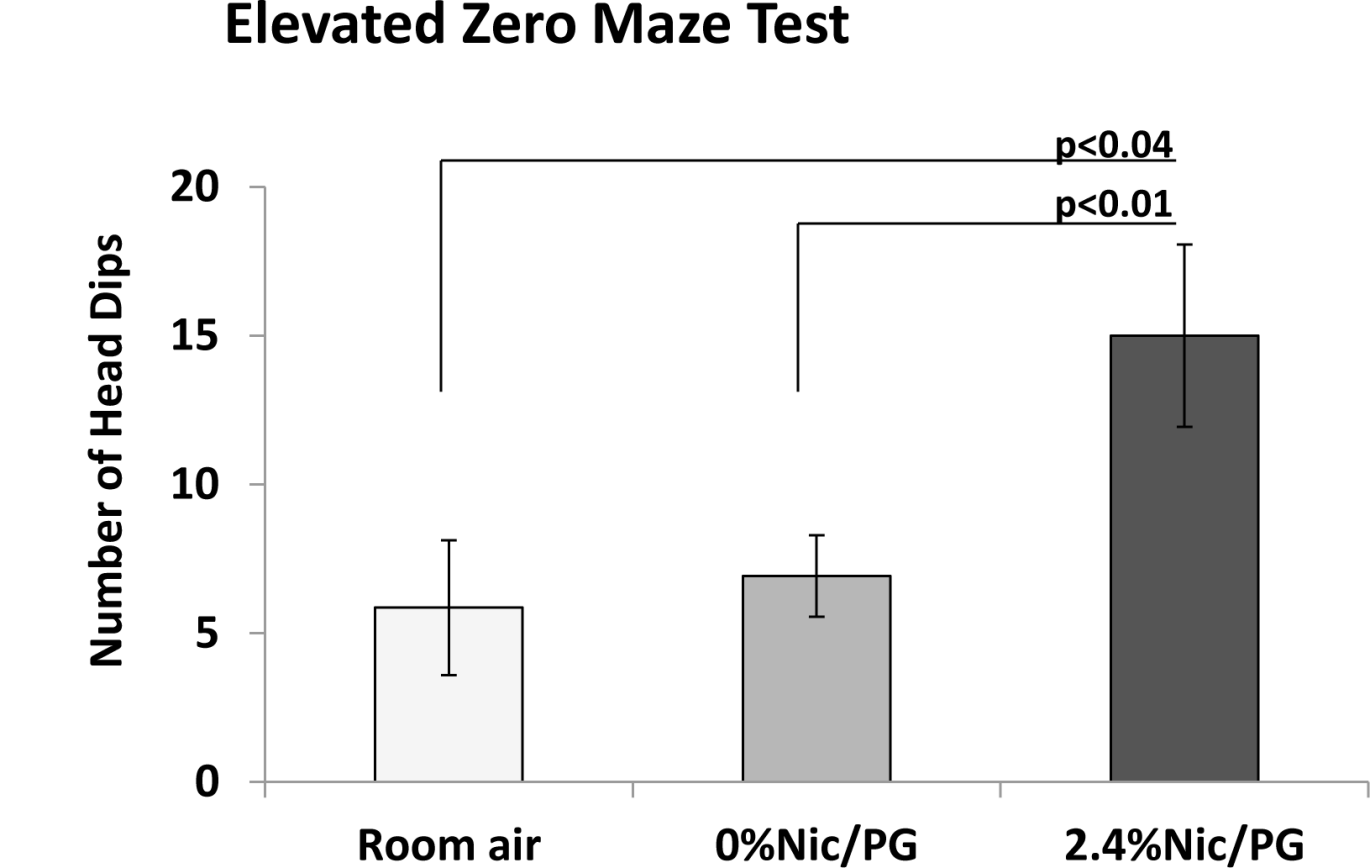
Elevated Zero Maze Test. Head dips were significantly greater in the 2.4% nicotine/PG mice compared to either the 0% nicotine/PG or the untreated mice (RA). (n = 7–13)

Anxiety-like behaviors were assessed on the elevated zero maze test and in the light/dark transition test. The time spent in open sections did not differ between the groups in the elevated zero maze test. Head dips, however, a measure of exploration [9], yielded a main effect of Treatment (F_(2,25)_ = 4.977, p = 0.015), (Fig 4). Follow-up analysis with *t*-test revealed that the 2.4% nicotine/PG mice had significantly more head dips compared to either the 0% nicotine/PG (p < 0.01) or the room air mice (p < 0.04). Data from the light-dark test showed that the 2.4% nicotine/PG mice tended to enter the lighted chamber more quickly (latency: ANOVA, p<0.53), and to spend more time in the lighted chamber than the other two groups, but these differences were not significant (duration: ANOVA, p<0.36).

**Fig 5:**
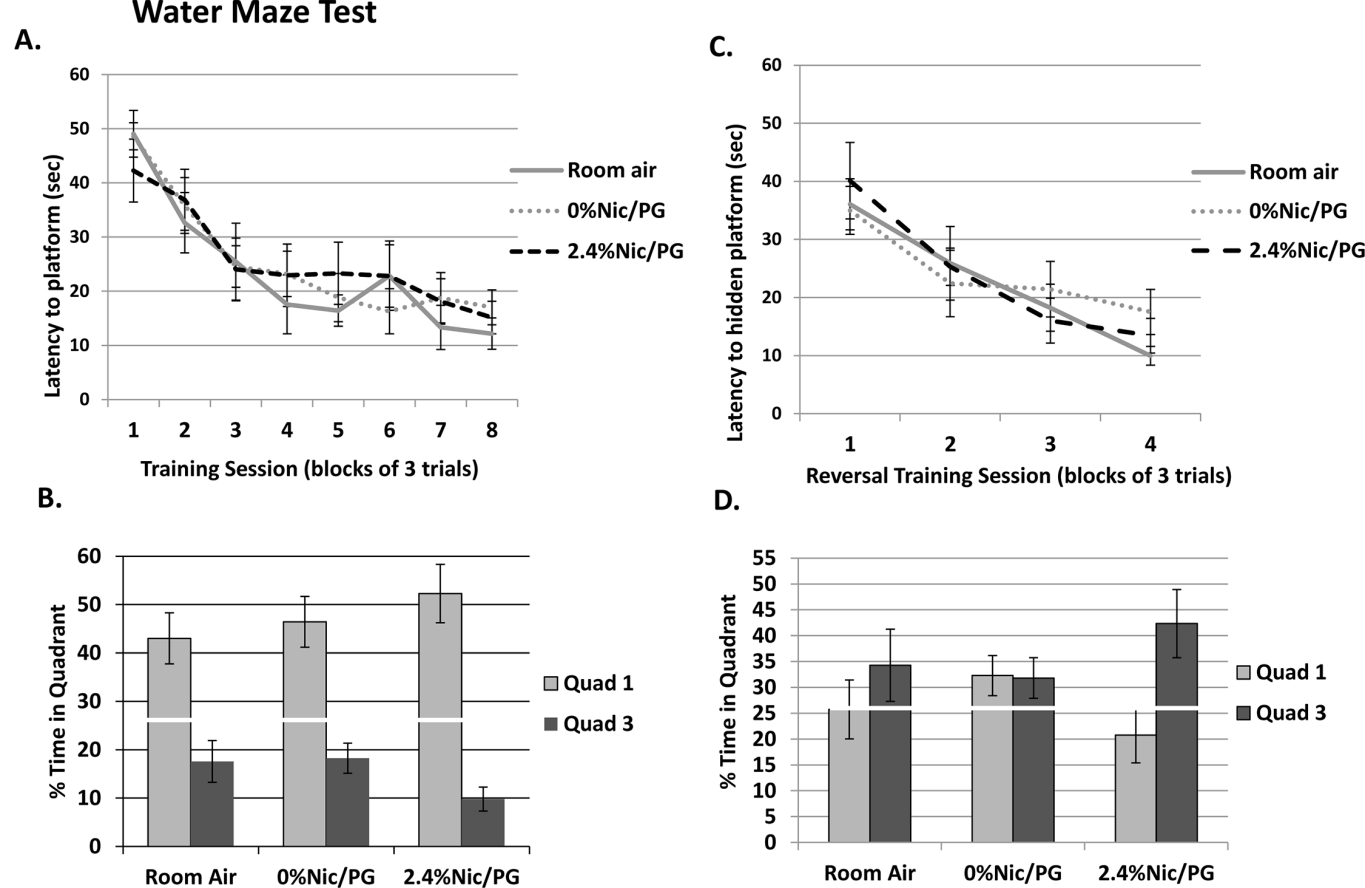
Water Maze testing with reversal learning. (A) Latency to the hidden platform during the initial place training phase. Over the course of training all three groups learned equally well to navigate to the hidden platform. (B) Illustration of data from trials in which the platform was unavailable. The figure shows the percent of time each group spent in the quadrant where the platform is normally located (Quad 1- target platform) versus the quadrant opposite of that (Quad 3). All three groups spent more time in the Target quadrant (Quad 1) relative to Quad 3 (p<0.001), (white line represents chance levels (25%). (C) Latency to locate hidden platform at new location in reversal training trials. Similar to training during initial place learning, all three groups decreased the time to the escape platform in a similar manner (p<0.001). (D) The percent time in each quadrant during the final probe trial after all training has been completed. In this trial the 2.4% nicotine/PG exposed mice trended towards a spatial bias for the new platform location, however analysis yielded only a marginal effect of Quadrant (F_1,25_ = 2.986, p < 0.096). The marginal effect prompted an analysis by one sample *t*-tests to compare each groups time in the new location to chance (25%), and only the 2.4% nicotine/PG exposed mice spent significantly more than 25% of time in the new location (t_7_ = 2.632, p < 0.034, n = 7–13).

To assess spatial learning and cognitive flexibility mice were trained to navigate to a hidden platform in a water maze, followed by reversal learning. Over the course of training in the initial phase, all three groups learned to navigate to the hidden platform equally well (Fig 5(A)) as confirmed by a significant main effect of Session (F_7,175_ = 23.283, p < 0.001) in the absence of a main effect of Treatment or a Session x Treatment interaction. Upon completion of place training, development of a spatial bias for the platform location was assessed in a probe trial. Spatial bias was measured as the percent time spent in the target quadrant vs. the opposite quadrant during the probe trial. All three treatment groups spent more time in the target quadrant relative to the opposite quadrant indicating that all mice developed a spatial bias for the target quadrant (Fig 5(B)). This was confirmed in a repeated measures ANOVA that yielded only a significant main effect of Quadrant (F _1,25_ = 40.724, p < 0.0001). There was no main effect of Treatment or a Quadrant x Treatment interaction.

Following the completion of place training all mice were given reversal learning training trials. In these trials the hidden escape platform was relocated to the quadrant opposite the one used during place learning. Fig 5(C) depicts the latencies for each group to reach the platform at the new location over the four training sessions. Similar to training during initial place learning, all three groups decreased the time to the escape platform over the course of training. As before, analysis yielded only a significant effect of Session (F_3,75_ = 29.567, p < 0.001), and there was no main effect of Treatment or a Session x Treatment interaction. To assess acquisition of spatial bias to the new platform location during reversal training, a probe trial was given to all mice after the first two sessions of training as well as after the completion of all reversal training (after 4 sessions of training). Spatial bias was again measured as the percent time spent in the target quadrant vs. the opposite quadrant. The results for the first probe trial interpolated after the second reversal training session demonstrated no significant effect of Quadrant (F_1,25_ = 0.611, p < 0.442) nor was there an effect of Treatment or Quadrant x Treatment interaction. Fig 5(D) depicts the percent time in each quadrant during the final probe trial after all training had been completed. In this trial the 2.4% nicotine/PG exposed mice trended towards a spatial bias for the new platform location, however analysis yielded only a marginal effect of Quadrant (F_1,25_ = 2.986, p < 0.096). The marginal effect prompted an analysis by one sample *t*-tests to compare each groups time in the new location to chance (25%), and only the 2.4% nicotine/PG exposed mice spent significantly more than 25% of time in the new location (t_7_ = 2.632, p < 0.034).

Finally, after all training and probe tests were completed, all mice were given cued trials in which the platform was visible to verify that none had any sensorimotor or visual defects that would interfere with performance in the water maze. Performance on cue training trials was measured by the mean latency to the visible platform (14.60 ± 6.61 for 2.4% nicotine mice, 15.47 ± 9.22 for 0% nicotine mice, 13.16 ± 6.49 for room air mice, overall). There were no significant treatment differences demonstrating that all groups were equally proficient at locating the visible platform (ANOVA, p<0.81).

To assess the robustness of our results, we performed an alternate analysis of our data using nested mixed models. [8] This allowed us to account for litter-based effects and to provide estimates of variance due to litter, (S1 Table. Estimates for the Percentage of Variation due to Litter-Related Effects). The results based on E-cigarette exposure found to be significant (*p*<0.05) by ANOVA testing were also significant by mixed modelling, (S1 Text. Analysis using Nested Mixed Modeling). The only substantial difference between the ANOVA-based analyses and the mixed model analyses was that for the total distance travelled in the open field test, the ANOVA test yielded only a significant main effect of Day (*p* = 0.035), whereas the mixed model did not demonstrate a significant main effect of Day (*p* = 0.35), but did show a main effect for Nicotine (*p* = 0.040).

## Discussion

In this study adult male mice previously exposed to E-cigarette nicotine vapors during late prenatal and early postnatal life, demonstrated increased levels of activity in the zero maze and open field test compared to mice exposed to E-cigarette vapors containing 0% nicotine and untreated controls. These findings suggest that nicotine exposure to E-cigarette vapors during a period of rapid brain growth can cause behavioral changes in adult male mice.

At the end of 14 days of postnatal exposure, juvenile mice exposed to 2.4% nicotine vapors had significantly higher serum cotinine levels compared to untreated or 0% nicotine vapor controls and significantly lower mean body weights than untreated controls. Interestingly the 0% E-cigarette vapor exposed juvenile mice had lower mean body weights than both untreated and 2.4% nicotine exposed mice. Although mothers and pups were only exposed to E-cigarette vapors for approximately 20 minutes a day, possible disruption in feeding or stress from exposure to E-cigarette vapors may have adversely affected weight compared to untreated controls. An association between attention deficit hyperactivity disorder and low intellectual performance has been reported in young adults born small for gestational age who were born at term. [10,11]

Nevertheless, we found that only mice exposed to 2.4% nicotine vapors and not 0% nicotine vapors had behavioral changes as adults compared to untreated controls. The 2.4% nicotine exposed mice were exposed to approximately 2.1 mg of nicotine/day, estimated from the daily amount of nicotine solution vaped into the chamber and adjusted for number of mice exposed in the chamber. Pups likely received additional nicotine exposure through breastmilk. A mean serum cotinine level of 23.7 ± 4.2 ng/ml was found in juvenile pups exposed to 2.4% nicotine vapors. Similar levels have been reported in newborns of mothers who smoked. Ivorra and colleagues measured plasma cotinine levels of 31.7 ± 8.6 ng/ml and 59 ± 13.3 ng/ml, 48 hours after birth, in newborns of mothers that reported moderate and heavy smoking. [12] In another study Chazeron and colleagues, reported a mean plasma cotinine level of 76 ng/ml in newborns of mothers who reported smoking.[13] Taken together our findings suggest that changes in adult behavior are associated with high nicotine exposure rather than lower total body weight caused by exposure to E-cigarette vapors.

No differences were noted between untreated controls and adult mice previously exposed to 0% nicotine containing vapors in any of the behavioral tests. However mice previously exposed to nicotine containing E-cigarette vapors had a significant increase in mean number of rearing and head dipping behaviors compared to untreated and 0% nicotine containing E-cigarette vapor controls. In the open field test, nicotine exposed mice also traveled longer distances during day one of testing compared to mice previously exposed to 0% nicotine/PG vapors. An increase in attention deficit hyperactivity disorder (ADHD) has been reported in offspring of mothers who smoked during pregnancy. [14,15] Previous studies in mice and rats exposed to prenatal and/or postnatal nicotine have also shown an increase in hyperactivity behavior, impaired inhibitory control and increased aggression. [16–20] [21,22] Findings from our study suggest that exposure during late prenatal and postnatal life to E-cigarette nicotine vapor can cause increased locomotor activity in young adult male mice.

In the water maze test followed by reversal learning the 2.4% nicotine exposed mice spent significantly more than 25% of time in the new location suggesting a tendency towards increased cognitive flexibility. Nicotine has been shown to improve cognition when administered acutely and has been investigated as a cognitive enhancer to mitigate cognitive deficits in diseases such as Alzheimer’s, schizophrenia and ADHD. [23] Studies in rodent models have showed mixed results with regard to nicotine enhancing cognitive flexibility. Schneider and colleagues exposed pregnant rats to nicotine containing water during gestation and reported less anxiety-related behavior, more anticipatory responses and fewer omission errors in the Five-Choice Serial Reaction-Time Task in adolescent offspring.[19] Alkam and colleagues exposed pregnant C57BL/6J mice to nicotine sweetened water during various gestational and early postnatal time points and found some deficits in working memory, object-based attention, and prepulse inhibition in offspring which was dependent on age during nicotine exposure. [24]

For analyses in this study, the statistical unit was an individual adult animal, rather than litter. This previously reported approach [25] [26] was used in our study to minimize the number of animals needed and because E-cigarette vapor exposure occurred predominately during postnatal life (4 days prenatal vs. 13 days postnatal), with behavioral studies performed in adult mice at 14 weeks of age. However, recognizing that the common environmental effects as encompassed by the litter may be a confounding factor with regards to the limited prenatal exposure in our study, we performed alternative analyses to account for litter effects using nested mixed models with random intercepts and slopes, and unstructured covariance.[8] Additionally, our nested mixed models also provide estimates of variance due to litter effects, which were generally minimal. We found that testing results related to exposure and identified as significant by the original ANOVA testing were also significant using mixed modeling. The exception was time spent in open sections of the elevated zero maze test in which the original ANOVA testing was not significant, whereas analysis performed by mixed modeling identified a significant effect for nicotine.

There were limitations to our study. Adult behavioral outcomes may differ depending on the developmental period of exposure, genetic background/species, sex and/or dose of nicotine used[27] and these variables were not explored in this study. In our study juvenile mice exposed to 0% nicotine containing vapors had low levels of serum cotinine, suggesting low dose nicotine contamination in the 0% E-cigarette solution used. Nevertheless the adult mice previously exposed to E-cigarette vapors containing 0% nicotine had similar behavioral testing as the untreated adult mice. Behavioral responses were also evaluated using a test battery and each animal was included in all behavioral assessments. This allowed for maximal collection of data from a limited sample. In addition since a previous behavioral test can impact later behavioral performance the order of testing was preformed from least to most invasive consistent with other investigators that have utilized test batteries [28] however we cannot know for certain whether no test order interactions exist. Finally chronic nicotine exposure during *in utero* and early postnatal life has been reported to alter density of nicotinic acetylcholine receptors (nAChR), decrease total brain DNA content and prematurely stimulate nAChRs. [29] Future studies are needed to determine the effect of nicotine from E-cigarette vapors and neurotransmitter expression and regulation in the developing brain.

In summary our findings indicate that exposure during late *in utero* and early postnatal life to E-cigarette vapors that contain nicotine can lead to behavioral changes in adult male mice.

## Supporting Information

S1 Table Estimates for the Percentage of Variation due to Litter-Related Effects. (DOCX)

S1 Text Analysis using Nested Mixed Modeling. (DOCX)
