# Supplementary material for: Adult Behavior in Male Mice Exposed to E-Cigarette Nicotine Vapors during Late Prenatal and Early Postnatal Life
Source: PLoS One. 2015 Sep 15;10(9):e0137953. doi: 10.1371/journal.pone.0137953 (PMC4570802; doi:10.1371/journal.pone.0137953)
Supplement: S1 Table — (DOCX) [file pone.0137953.s001.docx]

**S1 Table. Estimates for the Percentage of Variation due to Litter-Related Effects***

| **Test** | **Litter Variance** | **Total Variance** | **Variance due to Litter** |
| --- | --- | --- | --- |
| Rotarod | 20.37404 | 420.6133 | 4.8% |
| Open Field Test (Distance) | 117401.7 | 1105458 | 10.6% |
| Open Field Test (Rears) | 442.7185 | 1596.332 | 27.7% |
| Elevated Zero Maze (Open Sections) | 0.0000594 | 70544.65 | 0.0% |
| Elevated Zero Maze (Head Dips) | 8.026774 | 44.44503 | 18.1% |
| Light/Dark Transition Test (Latency) | 9.73E-11 | 24399.03 | 0.0% |
| Light/Dark Transition Test (Duration) | 1.43E-20 | 4521.321 | 0.0% |
| Water Maze (Training: Latency) | 42.87372 | 236.9533 | 18.1% |
| Water Maze (Training: Quadrant) | 1.92E-13 | 203.2066 | 0.0% |
| Water Maze (Reversal Training Phase: Latency) | 3.44E-12 | 341.8756 | 0.0% |
| Water Maze (Reversal Training Phase: Quadrant) | 2.26E-24 | 189.6634 | 0.0% |
| Water Maze (Final Probe Trial: Quadrant) | 1.96E-22 | 243.9908 | 0.0% |
| Water Maze (Cued Trials) | 1.83E-21 | 63.04396 | 0.0% |

*Variance estimates were taken directly from the raw STATA output. Variance due to litter was derived by dividing litter variance by total variance for the model.
