# Supplementary material for: Adult Behavior in Male Mice Exposed to E-Cigarette Nicotine Vapors during Late Prenatal and Early Postnatal Life
Source: PLoS One. 2015 Sep 15;10(9):e0137953. doi: 10.1371/journal.pone.0137953 (PMC4570802; doi:10.1371/journal.pone.0137953)
Supplement: S1 Text — (DOCX) [file pone.0137953.s002.docx]

**S1 Text. Analysis using Nested Mixed Modeling**

Recognizing that the common environmental effects as encompassed by the litter may be a confounding factor with regards to the limited prenatal exposure in our study, we performed alternative analyses as described below to account for litter effects. This was accomplished through the use of nested mixed models with random intercepts and slopes, and unstructured covariance. For all nested mixed models, individual animals were nested within their litters. For testing where multiple measurements were assessed for the same animal in sessions over time or on different days, measurements were nested within animals, which in turn were nested within litters; in these models, interactions between session and exposure or day and exposure were also included. Exposures were coded using two variables, namely exposure to the carrier (i.e., 0% or 2.4% nicotine) and exposure to nicotine (i.e., 2.4% nicotine) with each exposure having its own interaction term where appropriate. The results of our mixed model testing is outlined below. Furthermore, the use of mixed models allowed us to assess the variation for a given outcome attributable to litter-related effects, and these estimates are also provided below, which suggest that litter-related effects are negligible for most of the behavioral tests performed.

To summarize, testing results related to exposure identified as significant by the original ANOVA testing used in the original and revised manuscript were also significant using mixed modeling. The exception is time spent in open sections of the elevated zero maze test. In this case, the original ANOVA testing was not significant, whereas analysis performed by mixed modeling identified a significant effect for nicotine.

1. Rotarod: For latency to fall, the original ANOVA test yielded only a significant main effect of Day (*p*<0.001). The mixed model also yielded the same result (main effect Day: *p*=0.020).
2. Open Field Test (Distance): For total distance traveled, the original ANOVA test yielded only a significant main effect of Day (*p*=0.035) and t-tests for day 1 demonstrated that mice exposed to 2.4% nicotine/PG travelled significantly greater total distances than mice exposed to 0% nicotine (*p*<0.04) and tended to travel a greater distance than mice exposed to room air (*p*<0.09). The mixed model did not demonstrate a significant main effect of Day (*p*=0.35), but did show a main effect for Nicotine (*p*=0.040).
3. Open Field Test (Rears): For rearing behavior, the original ANOVA test yielded only a significant main effect of Treatment (*p*=0.003). The mixed model also yielded the same result (main effect Nicotine: *p*<0.001).
4. Elevated Zero Maze (Open Sections): For time spent in open sections, the original ANOVA test did not identify any significant effects. The mixed model demonstrated a significant main effect for Nicotine (*p*<0.001) and its interaction (Day x Nicotine: *p*=0.001).
5. Elevated Zero Maze (Head Dips): For number of head dips, the original ANOVA test yielded a significant main effect of Treatment (*p*=0.015). The mixed model also yielded the same result (main effect Nicotine: *p*=0.036).
6. Light/Dark Transition Test (Latency): For time to enter lighted chamber, the original ANOVA did not demonstrate significance for Treatment (*p*=0.53). The mixed model also yielded the same result (main effect Nicotine: *p*=0.48; main effect Carrier: *p*=0.57).
7. Light/Dark Transition Test (Duration): For duration of time spent in the lighted chamber, the original ANOVA did not demonstrate significance for Treatment (*p*=0.36). The mixed model also yielded the same result (main effect Nicotine: *p*=0.34; main effect Carrier: *p*=0.50).
8. Water Maze (Training Phase: Latency): For latency to platform, the original ANOVA demonstrated a main effect of Session (*p*<0.001). The mixed model also yielded the same result (main effect Session: *p*<0.001).
9. Water Maze (Training Phase: Quadrant): For time spent in the target quadrant compared to the opposite quadrant, the original ANOVA demonstrated a main effect of Quadrant (*p*<0.001). The mixed model also yielded the same result (main effect Quadrant: *p*=0.001).
10. Water Maze (Reversal Training Phase: Latency): For latency to the hidden platform, the original ANOVA demonstrated a main effect of Session (*p*<0.001). The mixed model also yielded the same result (main effect Session: *p*<0.001).
11. Water Maze (Reversal Training Phase: Quadrant): For time spent in the new target quadrant compared to the opposite quadrant, the original ANOVA did not demonstrate a main effect of Quadrant (*p*=0.442). The mixed model also yielded the same result (main effect Quadrant: *p*=0.215).
12. Water Maze (Final Probe Trial: Quadrant): For time spent in the target quadrant compared to the opposite quadrant, the original ANOVA suggested a trend for the main effect of Quadrant (*p*=0.096) with *t-*tests demonstrating that mice exposed to 2.4% nicotine spent more than 25% of time in the new location (*p*=0.034). The mixed model yielded a similar result (main effect Nicotine: *p*=0.100; Interaction Day x Nicotine: *p*=0.026).
13. Water Maze (Cued Trials): For latency to the visible platform, the original ANOVA did not demonstrate significance for Treatment (*p*=0.81). The mixed model also yielded the same result (main effect Nicotine: *p*=0.52; main effect Carrier: *p*=0.82).
